# Unraveling the *Bombus terrestris* Hemolymph, an Indicator of the Immune Response to Microbial Infections, through Complementary Mass Spectrometry Approaches

**DOI:** 10.3390/ijms24054658

**Published:** 2023-02-28

**Authors:** Lorène Bournonville, Dalel Askri, Karim Arafah, Sébastien N. Voisin, Michel Bocquet, Philippe Bulet

**Affiliations:** 1 Platform BioPark Archamps, 218 Avenue Marie Curie ArchParc, 74160 Archamps, France; 2 Department of Molecular and Cellular Biology, University of Geneva, Sciences III, 30 Quai Ernest-Ansermet, 1211 Geneva, Switzerland; 3 Phylogene S.A. 62 RN113, 30620 Bernis, France; 4 Michel Bocquet, Apimedia, 82 Route de Proméry, Pringy, 74370 Annecy, France; 5 Institute for Advanced Biosciences, Inserm U 1209, CNRS UMR 5309, University Grenoble Alpes, 38000 Grenoble, France

**Keywords:** bumble bee, *Bombus terrestris*, immune response, bee health, pathogens, proteomics, molecular mass fingerprints

## Abstract

Pollinators, including *Bombus terrestris*, are crucial for maintaining biodiversity in ecosystems and for agriculture. Deciphering their immune response under stress conditions is a key issue for protecting these populations. To assess this metric, we analyzed the *B. terrestris* hemolymph as an indicator of their immune status. Hemolymph analysis was carried out using mass spectrometry, MALDI molecular mass fingerprinting was used for its effectiveness in assessing the immune status, and high-resolution mass spectrometry was used to measure the impact of experimental bacterial infections on the “hemoproteome”. By infecting with three different types of bacteria, we observed that *B. terrestris* reacts in a specific way to bacterial attacks. Indeed, bacteria impact survival and stimulate an immune response in infected individuals, visible through changes in the molecular composition of their hemolymph. The characterization and label-free quantification of proteins involved in specific signaling pathways in bumble bees by bottom-up proteomics revealed differences in protein expression between the non-experimentally infected and the infected bees. Our results highlight the alteration of pathways involved in immune and defense reactions, stress, and energetic metabolism. Lastly, we developed molecular signatures reflecting the health status of *B. terrestris* to pave the way for diagnosis/prognosis tools in response to environmental stress.

## 1. Introduction

Bees are fundamental insects in agroecosystems, mainly due to their role in pollination. They are of crucial importance for the diversity of ecosystems and agriculture. Overall, more than one-third (35%) of the world’s food production is pollinated by bees, including bumble bees [1]. According to a report by the Intergovernmental Science-Policy Platform on Biodiversity and Ecosystem Services [2], this represents between 5 and 8% of the value of global food production. Unfortunately declines in pollinator populations have been documented since 2006, with suggestions that 40% of insect species worldwide are at risk of extinction in the coming decades, thus resulting in an overall loss of biodiversity on a planetary scale [3]. While the use of chemical molecules such as insecticides, herbicides, fungicides, and acaricides has contributed to advances in world agriculture [4], their potential negative impact on pollinators needs to be considered. After honey bees, bumble bees are the second-most economically important bee pollinator species worldwide. The bumble bee *Bombus terrestris* is one of the most common wild Eurasian pollinators. Its ability to forage at low temperatures and to vibrate the flowers make it a very efficient pollinator, even more so than other bees [5]. This technique is particularly suited to flowers in the *Solanaceae* family, such as tomatoes. Their pollination efficiency has led to significant commercial breeding of these populations, especially for the production of dozens of greenhouse crops [6]. Nevertheless, the significant decline in these populations among noncommercial bumble bee colonies of various species is concerning. There are various causes for this decline, including the reduction in their natural habitats and nutritional resources, global warming, the use of agricultural pesticides, and the presence of infectious agents (e.g., viruses, bacteria, fungi, and parasites) naturally present in the environment [7,8]. To fight these pathogens, bumble bees, similar to all metazoans, have developed defense mechanisms that rely heavily on innate immune responses [9,10]. Innate immunity, which represents the first line of defense shared by all living organisms (plants, invertebrates, and vertebrates) against harmful stressors and pathogenic invaders, encompasses virtually all tissues [11]. Among these tissues, plant sap, vertebrate blood, and insect hemolymph are the centers of the humoral immune response, which involves clotting, melanin production, and synthesis of immune effectors, such as antimicrobial peptides (AMPs), that target pathogens [12,13]. Insects, and particularly the genetic workhorse biological model, *Drosophila melanogaster*, have been studied to decipher innate immunity [10]. One of the key findings to emerge since the 2000s is the observation that AMP production is more exclusive than initially thought and varies depending on the type of infection [11,14].

For Hymenoptera, despite a fragmented scientific understanding of their innate immune responses, several studies have better characterized the antimicrobial response of *Apis mellifera* and the bumble bee *B. terrestris* thanks to genome sequencing programs. The *A. mellifera* immune system is quite similar to that of *D. melanogaster* and *Anopheles* mosquitoes, with one-third of the immune genes shared by the fruit fly and the mosquito [8,9]. Interestingly, as a social insect, *A. mellifera* has more genes for olfactory receptors and genes involved in the regulation of pollen and nectar collection [15]. In their hemolymph, insects secrete AMPs that are involved in the activated innate immune response to microbial infections [16]. Bees such as bumble bees mainly secrete four AMPs (apidaecin, abaecin, defensin, and hymenoptaecin) that provide a broad-spectrum antibacterial defense [17]. The molecular understanding of the innate immune response and its primary components, the AMPs, remains poorly documented. At the individual level, bacterial infection can induce metabolic deregulations, particularly in biological pathways involved in energy management, stress response, or defense mechanisms [18]. For several years, much work, especially in proteomics, has been undertaken on *A. mellifera*. These studies focused on the proteomic changes at different life stages of the honey bee using the hemolymph as a matrix [19,20,21,22,23,24,25]. Most of the proteomic studies have been performed to study the impact of the parasite *Varroa destructor* [22,24,25,26]. Proteomic analyses were also performed to distinguish between honey bee strains [21,23], castes [19], or season [27]. Most of the methods used in the above-mentioned studies were gel-based. It is now well documented that several immune defense reactions take place in the hemolymph of bees, such as the phagocytosis, melanization, coagulation, or secretion of AMPs produced by hemocytes and the fat body (the functional equivalent of the mammalian liver) [28]. The main goal of our work was to trigger an effective immune response in the bumble bee to different bacterial stressors to assess the impact of those biotic stressors on the health status of the bumble bee at the molecular level. This study followed a similar study performed on *A. mellifera* using different bacterial strains that may be challenging for bees [29]. We focused on the Gram-positive bacterial species *Micrococcus luteus*, which is widely distributed in the environment in insects, and two Gram-negative entomopathogenic species, *Pectobacterium carotovorum* subsp. *carotovorum* [29] and the opportunistic pathogen of bees *Serratia marcescens* [30]. We observed the increasing mortality of bumble bees, giving a gradual response to bacterial aggression. Using complementary mass spectrometry (MS) approaches (matrix-assisted laser desorption ionization-time of flight, MALDI-TOF) and high-performance liquid chromatography coupled to high-resolution tandem electrospray MS (LC-ESI-MS/MS), we observed different molecular mass profiles of the bumble bee hemolymph in response to the different bacterial strains used as biotic stressors and highlighted differences in the physiological pathways impacted by the challenging bacterial strain of interest.

## 2. Results and Discussion

Deciphering the overall humoral immune responses of the bumble bee *Bombus terrestris* at the molecular level is essential for a comprehensive understanding of how the bumble bee is impacted by stressors (abiotic and/or biotic). We designed an experimental infection workflow to evaluate by mass spectrometry (MS) the biochemical/physiological changes occurring in the bumble bee hemolymph. This tissue was collected after experimental infections using a needle of 0.1 mm diameter with two opportunistic entomopathogenic bacteria, *Pectobacterium carotovorum* subsp. *carotovorum* 15 (*P. c. c*.) and *Serratia marcescens* (*S. m*.), and an opportunistic non-pathogenic strain widely distributed in nature, namely *Micrococcus luteus* (*M. l.*).

### 2.1. Mortality Rate of Experimentally Infected Bumble Bees Versus Control

**Figure 1:**
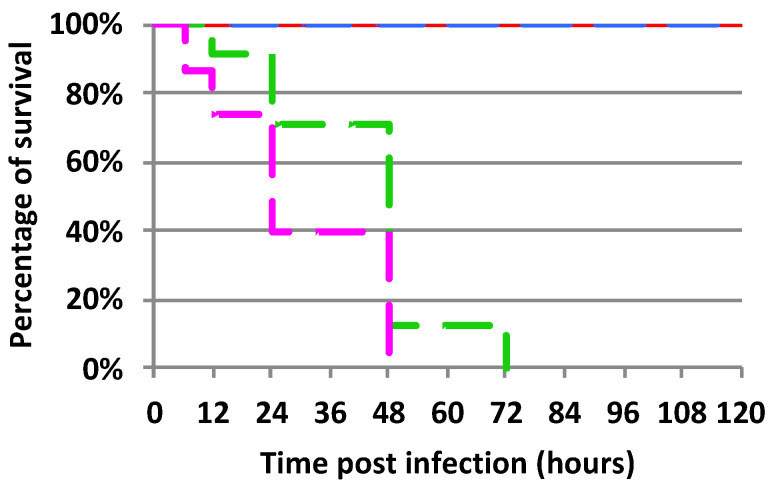
Survival of *Bombus terrestris* after infections with *Micrococcus luteus* (blue), *Pectobacterium carotovorum* subsp. *carotovorum* (green), and *Serratia marcescens* (pink). The control experiment (non-experimentally infected bees) is reported in red.

We compared the mortality rate of bacteria-inoculated bumble bees to that of controls (non-experimentally infected). Our results showed an effect on bumble bees regardless of the bacterial strain used in our experimental infections (Figure 1). Statistical analyses performed on the survival rate of *B. terrestris* after infection with different bacterial strains showed that both entomopathogenic Gram-negative bacteria used for experimental infections were pathogenic to bumble bees, *P. c. c.* strain (test log-rank; χ2 = 49.94; *p*-value < 0.0001) and *S. m.* strain (test log-rank; χ2 = 50.25; *p*-value < 0.0001). *S. m.* induced slightly higher mortality than *P. c. c.* (test log-rank; χ2 = 7.47; *p*-value < 0.01). Since infection with *S. m*. was performed by pricking the bumble bee with a fine needle dipped into a bacterial pellet, the experimental methodology must be considered when interpreting these results. It would be interesting to perform a titration of the number of bacteria inoculated rather than simply pricking individuals with a septic needle. Since *S. m.* is lethal to bees [30], a larger number of infected individuals would be required. In contrast, infection with the Gram-positive bacteria *M. l.* did not cause mortality in bumble bees when compared to the control condition in which bumble bees were not experimentally infected. As previously observed for *Apis mellifera* (personal observation P. Bulet), *M. l.* appears to be of low pathogenicity for *B. terrestris*. These first results indicated that *P. c. c.* and *S. m.* impacted the health status of *B. terrestris* and that *M. l.* did not induce mortality during the time course of our experiment (up to 5 days). Our results showed that bacterial infection may have small, mild, or deleterious effects on bumble bee individuals. 

### 2.2. Molecular Hemolymph Signatures by MALDI BeeTyping^®^ to Follow the Impact of Bacterial Infections in B. terrestris

To evaluate the impact of these experimental infections on the peptidomics/proteomics level, individual hemolymph samples were collected at different times after experimental infection (24 h, 48 h, and up to 5 and 15 days when possible) and the molecular mass fingerprints (MFPs) were recorded using MALDI mass spectrometry (referred as MALDI BeeTyping^®^).

MFPs by MALDI MS are a commonly used strategy to elucidate molecular signatures of complex biological matrices such as tissue extracts or direct body fluids under different physiological conditions (e.g., diseases, response to treatments, or stressors). To our knowledge, this approach has not yet been used to follow the immune status of the bumble bee *B. terrestris*, despite it already being used to monitor the immune responses of other insects (e.g., *Drosophila*) [31] to different biotic stressors. The use of MALDI MFPs was pioneered on an insect model, the fruit fly *Drosophila melanogaster*, in the late 1990s [31,32,33]. Through MALDI MFP analyses, the authors showed that this method applied at the individual scale provided reliable molecular mass signatures of *Drosophila* immune-induced molecules (DIMs), including four AMPs (two glycosylated forms of drosocins, metchnikowin, and the antifungal drosomycin) detectable in the mass range 1.5 to 11 kDa [33]. Interestingly, in addition to the AMPs identified by their molecular masses, a series of highly infection-induced peptides, the *Drosophila*-specific Bomanin peptides (Boms former DIMS 1-4), were recognized as critical for resistance against pathogens [34,35]. 

In this study, MFPs were used to follow the impact of experimental microbial infections in the bumble bee hemolymph according to a procedure applied to the honey bee, *A. mellifera* [29,36], and the honey bee pathogen, *Nosema* [35]. 

#### 2.2.1. Global Analysis of MFPs by MALDI BeeTyping^®^ Discriminates between Different Bacterial Infections

**Figure 2:**
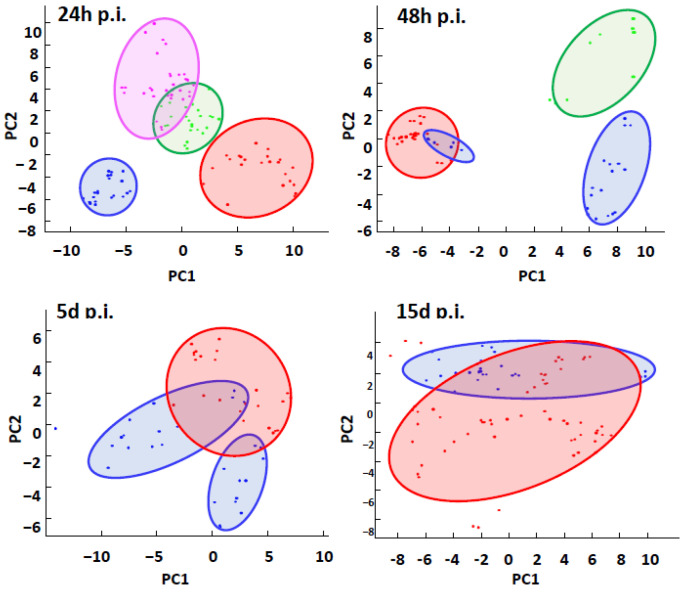
Principal component analysis (PCA) of the MALDI BeeTyping^®^ MFPs of the hemolymph of bumble bees infected with different bacteria strains during different exposure times (24 h post experimental infection (24 h p.i.); 48 h p.i.; 5 days p.i. (5 d p.i.) and 15 d p.i.). Each individual was analyzed in three replicates, i.e., three spectra represented by three points. The MFPs of each experimental condition were subjected to PCA to identify potential discrimination between the control group (red) and the different bacterial infections: *Micrococcus luteus* (blue), *Pectobacterium carotovorum* subsp. *carotovorum* (green), and *Serratia marcescens* (magenta).

To generate MFP models, an average hemolymph spectrum was recorded as the signature of this tissue in response to each pathogen used (*M. l.*, *P. c. c.*, or *S. m.*). Statistical principal component analysis (PCA) (Figure 2) showed that the three infected groups differed from the control group 24 h post-experimental infection (24 h p.i.). Over time, the group infected with the Gram-positive strain *M. l.* appears to be less distinguishable from the control group, in contrast to the group infected with *P. c. c.*, which can still clearly be distinguished at 48 h p.i. After 5 d p.i., the group infected with *M. luteus* tends to overlap with the control group, while after 15 days, the *M. l.* infected group was no longer distinguishable from the control one. As already evidenced by our survival follow-up, none of the experimentally infected bumble bees could survive being pricked with a fine needle dipped into a bacterial pellet of *S. m.*

Overall, this analysis showed that within 24 h p.i, MFPs reflected the impact of the different strains tested, from a widely distributed non-pathogenic strain (*M. l.*) to an opportunistic strain with a high pathogenicity, such as *S. m.*, to be discriminated. This reflects the impact of the strains on the molecular complexity of the hemolymph and represents a read-out of the humoral immune response of the bumble bee. 

#### 2.2.2. The Immune Response to Microbial Infections

**Figure 3:**
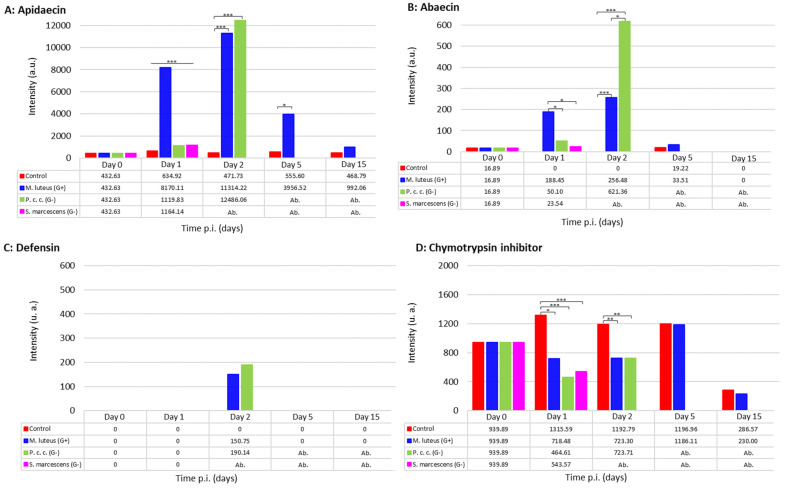
Average intensity of some immune molecules in bumble bee hemolymph, apidaecin (**A**), abaecin (**B**), defensin (**C**), and chymotrypsin inhibitor (**D**). The intensity averages were analyzed using Kruskal–Wallis statistical tests with significant differences at *p*-value < 0.05 (*), highly significant at *p*-value < 0.01 (**), and very highly significant at *p*-value < 0.001 (***).

To further understand the defense responses of bumble bees against microbial infections at the molecular level, we looked for the presence of antimicrobial peptides (AMPs) in the hemolymph of the bees in response to bacterial infections. Indeed, in response to pathogens, insects secrete a series of defense molecules in their hemolymph to generate an immune response capable of dealing with different infections [10,37]. AMPs are key players among these defense molecules. In bumble bees, several AMPs have already been characterized, apidaecin (accession C0HKX3), abaecin (accession D2XR04), hymenoptaecin (accession D2XR06), and defensin 1 (accession D2XR05). We followed the dynamics of AMP expression during various bacterial challenges. Peak intensities of apidaecin (Figure 3A) and abaecin (Figure 3B) were measured 48 h p.i. in bumble bees infected with *M. l.* and *P. c. c.* (*p*-value < 0.0001). A significant (*p*-value < 0.0001) increase in the intensity of both peaks is visible only 24 h after infection with *M. l.* Defensin 1 was only visible in the *M. l.* and *P. c. c.* groups after 48 h p.i. at a rather low level (Figure 3C). Erler and colleagues reported that gene expressions of *defensin 1* and *hymenoptaecin* were significantly linked to bacterial growth in *B. terrestris* over 24 h [18]. They also observed that for *defensin 1*, the pattern of gene expression in response to *Escherichia coli* infection started later, at 8 h p.i., compared to high expression of the *abaecin* and *hymenoptaecin* genes as early as 4 h p.i. Under our experimental conditions, hymenoptaecin was not detected or was below the signal to noise ratio. Such results contrast with our observations that when using the pathogenic microorganism *S. m.*, bumble bee survival was profoundly affected (Figure 1) and the immune response was rapidly overwhelmed by infectious events, with AMPs detected 24 h p.i. *S. m.* is an environmental bacterium that acts as a nosocomial pathogen of humans and an opportunistic pathogen of insects including *A. mellifera* [38,39]. The virulence of this bacterium is too strong for the bumble bees to defend themselves and activate their immunity. In general, its virulence is partly attributed to the virulence factor Serralysin [40,41]. In addition, Haddix and collaborators reported that the red pigment prodigiosin synthesized by *S. m*. increases the multiplication rate of the bacterium [42]. 

In addition to the above AMPs, we followed the intensity of the expression of the chymotrypsin inhibitor (Figure 3D, accession K7WRE1), a serine-type endopeptidase inhibitor circulating in bee hemolymph. This protein was found to be deregulated during different infections at 24 and 48 h p.i., and a deregulation was observed in *A. mellifera* by Houdelet and colleagues in response to experimental infection with *Nosema* spores [43].

According to our results, infection with *M. l.* triggers an immune response more rapidly than an infection with *P. c. c.*, which would allow bumble bees to effectively defend themselves against *M. l.*, thus blocking its multiplication. *P. c. c.* appears to strongly stimulate the immune system of bumble bees but does so too late, which would explain the relatively high mortality rate observed previously (Figure 1).

### 2.3. Development of a Computational Model to Discriminate between the Stressor and Its Impact

The MFPs recorded by MALDI BeeTyping^®^ were used to develop predictive models that could discriminate molecular responses to the microbial infections presented in this study based on machine learning algorithms.

**Figure 4:**
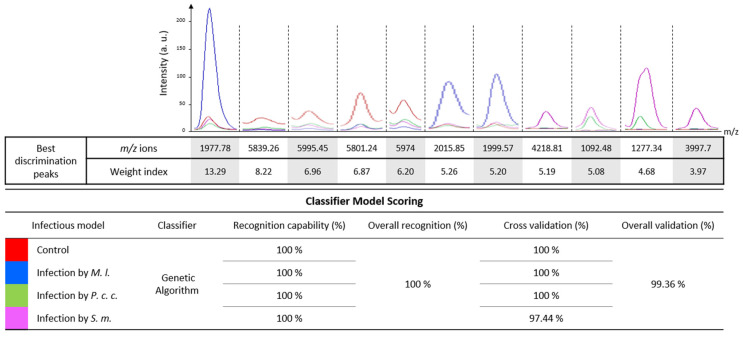
Biological models for the classification of uninfected or experimentally infected bumble bees. These models were generated from eleven molecular ion peaks selected according to their importance index to have the best discriminating characteristics of hemolymph samples. They were obtained by an algorithmic program of the ClinProTools™ software configured with a maximum of 50 generations and a maximum selection of 15 peaks. Only the spectra obtained at 24 h p.i. were considered to generate the models. A classifier based on a genetic algorithm was used to distinguish between control (non-experimentally infected, red) and experimentally infected bees: *Micrococcus luteus* (*M. l*., blue), *Pectobacterium carotovorum* subsp. *carotovorum* (*P. c. c*., green), and *Serratia marcescens* (*S. m*., magenta).

Control/non-experimentally or experimentally infected bumble bee samples were classified using a set of eleven molecular ions that were found to discriminate the different biological models (Figure 4). From the MALDI MFPs, the genetic algorithm-based models recognized each biological condition with 100% accuracy. Cross-validation of the different individual hemolymph MFPs resulted in a 100% match for the control model, *M. l.*, and *P. c. c.* and a 97.44% match for the *S. m.* experimental infection model (Figure 4). The most discriminating molecular ion (*m*/*z* 1977.78) was apidaecin (theoretical *m*/*z* = 1978), in agreement with the results found in Figure 3.

Based on their respective MFPs, all four experimental conditions exhibited a good recognition score based on a small number of molecular ions. At this stage, we are unable to identify whether these ions originate from the bacteria or reflect the impact of the bacteria on the bumble bee hemolymph composition. This result confirms similar results obtained by Arafah et al. [29], and paves the way for using MALDI BeeTyping^®^ as a diagnostic tool to evaluate the health status of *Bombus*. Further experiments are needed to confirm these results with bumble bees of other origins to verify the robustness of the model and its applicability to a wider population range. These MALDI MS analyses revealed the impact of bacterial infections on the molecular composition of bumble bee hemolymph, particularly on certain immune peptides. Unfortunately, a limitation of this technique is that no structural characterization was possible in our experimental conditions. This technique alone is not sufficient to characterize the pathways that are altered in response to the bacterial infections considered in this study. Some molecules are not or are barely detectable in the mass range studied by MALDI BeeTyping^®^ (1 to 18 kDa). To circumvent this limitation and to characterize proteins over 18 kDa that might be deregulated in response to the different bacterial infections, we performed off-gel bottom-up proteomics analyses using high-performance liquid chromatography coupled to high-resolution tandem electrospray MS (LC-ESI-MS/MS).

### 2.4. Proteomics Analysis of the Hemolymph of B. terrestris in the Context of Experimental Bacterial Infections

**Figure 5:**
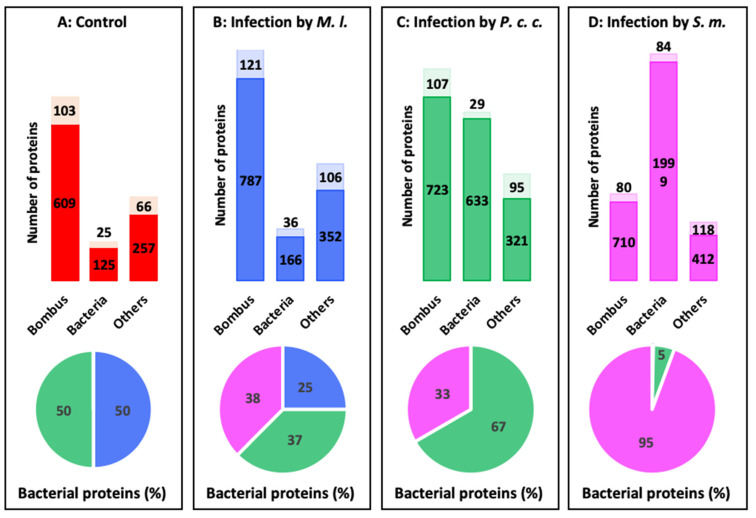
Overall number of proteins identified in the hemolymph of *Bombus terrestris* by off-gel digestion and LC-ESI-MS/MS at 24 h post-infection. All identified proteins were obtained after proteomic analysis of the different experimental conditions in control (**A**), bumble bees infected with *Micrococcus luteus* ((**B**), *M. l.*), *Pectobacterium carotovorum* subsp. *carotovorum* ((**C**): *P. c. c.*), and *Serratia marcescens* ((**D**): *S. m.*). For each condition, the proportions of identifications of proteins from the genus *Bombus*, bacteria, and others are plotted in the bar charts. The number of characterized and uncharacterized proteins are in dark and light colors, respectively. The (**A**–**D**) pie charts are representative of the percentage of bacterial proteins originating from control (non-experimentally infected, red), *M. l.* (blue), *P. c. c.* (green), and *S. m.* (magenta).

**Figure 6:**
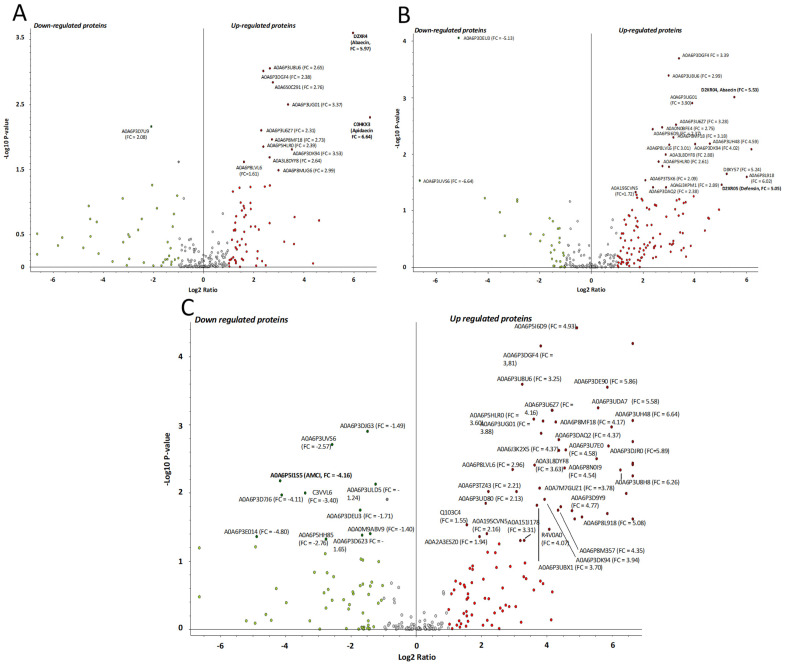
Up- and down-regulated proteins in bumble bee hemolymph following an experimental bacterial challenge with *Micrococcus luteus* (**A**), *Pectobacterium carotovorum* subsp. *carotovorum* (**B**), or *S. marcescens* (**C**) versus the control experiment (non-experimentally infected bumble bees). Dots colored in red represent proteins with a fold change of >2 (up-regulation threshold), while the ones in green are for a fold change of <−2 (down-regulation). The dots close to the red and green boxes represent the proteins (denoted by their UniProtKB accession number) that were found to be significantly up-regulated (FC > 2, *p*-value < 0.05) and down-regulated (FC < −2, *p*-value < 0.05) in response to the infection, respectively. Gray dots represent all remaining proteins that did not pass the thresholds −2 > FC > 2 and the *p*-value < 0.05 criterium.

From the bottom-up proteomics analysis by off-gel digestion and LC-ESI-MS/MS on bumble bee hemolymph samples, we identified a total of 1185 proteins in the control condition (Figure 5A), 1568 during the experimental infection with *M. l.* (Figure 5B), 1908 upon infection with *P. c. c.* (Figure 5C), and 3403 upon infection with the entomopathogenic *S. m.* (Figure 5D). The majority of these proteins were attributed to the genus *Bombus*. Among these proteins, a large majority (Figure 5A–D; values expressed in %) were characterized (dark colors in Figure 5), while a few of them (light colors in Figure 6) were still classified as uncharacterized due to partial availability of genome annotation for *B. terrestris* [18]. During infection with *P. c. c.* and *S. m.*, a significant number of bacterial proteins were identified in the hemolymph samples, precisely 96 after the experimental challenge with *P. c. c.* (64 from *P. c. c.* and 32 from *S. m.*). Interestingly, with the most pathogenic strain *S. marcescens* (Figure 1), 1120 out of 3403 proteins were found to be from *Serratia* origin, and 63 proteins from *Pectobacterium*. This contrasts with the extremely low number of bacterial proteins (eight) detected in the hemolymph samples of bumble bees infected with *M. l.* (Table S1). We hypothesize that the bumble bees belonging to the *M. l.* group can develop an immune response effective enough to neutralize the intruder. This is in part attributed to the secretion of immune defense molecules, such as apidaecin, abaecin, and defensin 1 (Figure 3A–C), and AMPs with complementary activity spectra [44,45]. Several additional identified proteins were attributed to non-bumble bee hymenopterans (1313, Table S1) and non-bacterial pathogens (3 from viruses, 143 from fungi, and 195 from parasites). The characterization of bacterial proteins from the two strains that mildly (*P. c. c.*) or strongly (*S. m.*) affect bumble bee survival suggests that these bacteria evaded the immune response of bumble bees and were able to multiply within the haemocoel.

The phytopathogenic *P. c. c.* is a Gram-negative bacterial strain of the *Enterobacteriaceae* family. This family causes various diseases in plants, a pathogenicity due to the production of virulent factors [46]. Such pathogenetic bacteria have developed plant-to-plant infection cycles, often via insects, including bees [47,48]. In the insect model *Drosophila melanogaster*, *P. c. c.* infections induced an antimicrobial response in larvae with a dominant expression of antibacterial vs. antifungal peptide-encoding genes [49]. The honey bee was also reported to be a vector of *Erwinia amylovora* [50], which can survive in the honey bee body for several days [51]. In our experimental conditions, the presence of several proteins from a *P. c. c.* origin suggests that this bacterium is only partially controlled by the immune responses of the bumble bee, at least within the first three days of the infection. The sharp increase in the mortality rate observed after 48 h (Figure 1) indicates that *P. c. c.* is pathogenic to the bumble bee and represents a risk for its survival.

### 2.5. Treatment-Dependent Protein Intensity

Following bacterial challenge with either *M. l.*, *P. c. c.*, or *S. m.*, label-free quantitative (LFQ) proteomics were conducted on pools of hemolymph. Fold-change ratios for 538 proteins were calculated as a measurement of protein regulation in infectious versus control conditions (Table S2). Amongst the 13 proteins from bumble bees and other hymenopterans (Figure 6A and Table S3) which were found to be significantly up-regulated in the *M. l.* infected group, abaecin (FC = 5.97) and apidaecin (FC = 6.64) exhibited the highest fold change, as already observed on the molecular signatures obtained by MALDI BeeTyping^®^ (see Figure 4). An uncharacterized protein (accession A0A6PD7U9, FC = −2.08) was found to be significantly down-regulated during the *M. l.* challenge. Twenty-two proteins from *B. terrestris* and other hymenopteran species were significantly up-regulated in bumble bees infected with *P. c. c.* (Figure 6B and Table S3), among them were abaecin (FC = 5.54) and defensin 1 (FC = 5.05), thus confirming the results of MALDI BeeTyping^®^ (Figure 4). In addition, two proteins from the *Paenibacillus* species (accession A0A7X3FGB9, FC = 6.21 and A0A1G8J1H5, FC = 3.01) and one from *S. m.* (A0A0J5D3A5, FC = 2.76) were found to be significantly up-regulated (Table S2). Two proteins (A0A6P3DEU3, FC = −5.13 and A0A6P3UVS6, FC = −6.64) from *B. terrestris* and *B. impatiens* and other proteins matching hymenopteran species were found to be down-regulated (Figure 6B). The bacterial infection of bumble bees with the entomopathogenic bacterial strain *S. m.* resulted in numerous proteins with significantly altered abundance (Figure 6C and Table S3). Forty-four proteins were up-regulated and six were down-regulated, including the chymotrypsin inhibitor (AMCI, FC = −4.17). Based on the number of dysregulated proteins quantified in the proteomics study following each type of infection, we hypothesized that *S. m.* is of higher pathogenicity than *P. c. c.* and *M. l.* With the latter, the *B. terrestris* proteome was not disturbed as with the two first strains. This is in line with what we observed following the mortality and MALDI BeeTyping^®^ study.

**Figure 7:**
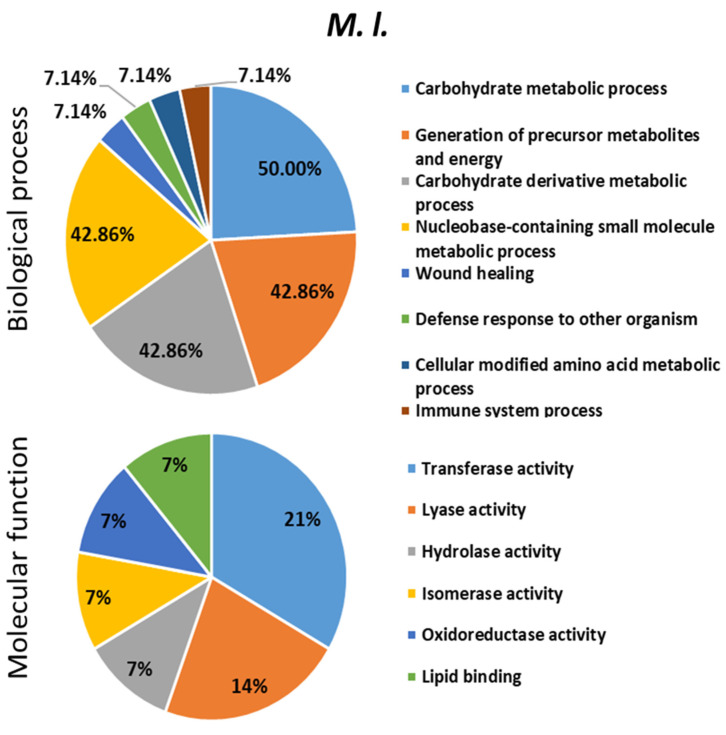
Biological processes and molecular functions of the dysregulated proteins evidenced following *B. terrestris* exposure to the bacteria *Micrococcus luteus* (*M. l.).* The percentage was calculated by dividing the number of the proteins found in each biological process or molecular function identified with OmicsBox software by the number of the total dysregulated proteins following bacterial challenge. The pie-charts were made with Microsoft Excel 2021.

**Figure 8:**
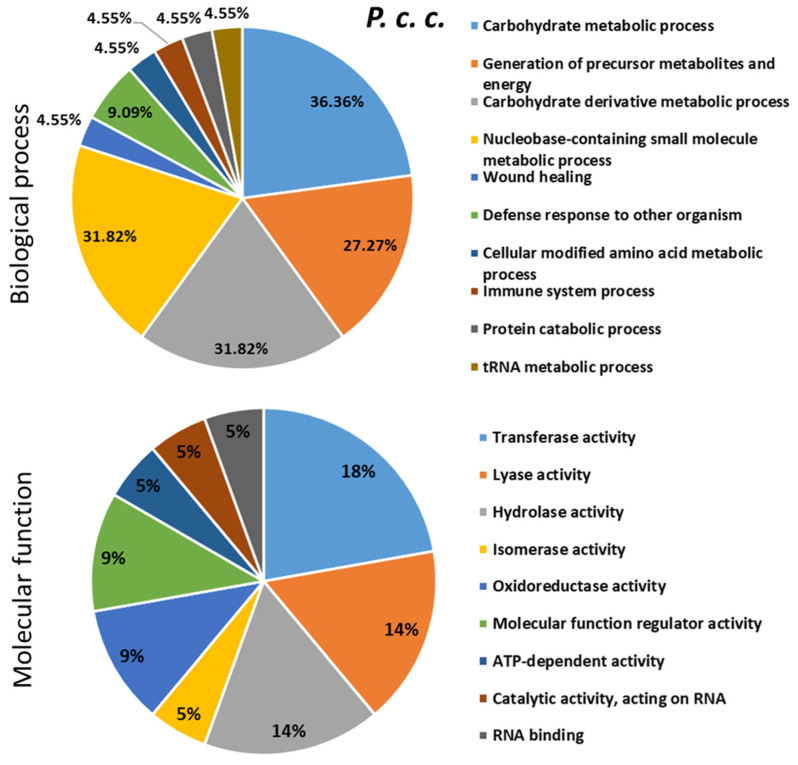
Biological processes and molecular functions of the dysregulated proteins evidenced following *B. terrestris* exposure to the bacteria *Pectobacterium carotovorum* subsp. *carotovorum (P. c. c.).* The percentage was calculated by dividing the number of the proteins found in each biological process or molecular function identified with OmicsBox software by the number of the total dysregulated proteins following bacterial challenge. The pie-charts were made with Microsoft Excel 2021.

**Figure 9:**
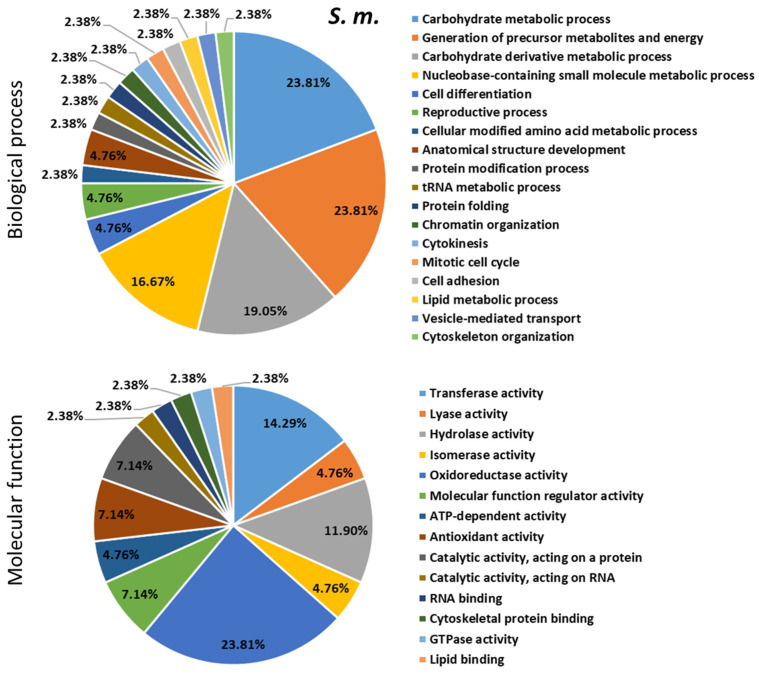
Biological processes and molecular functions of the dysregulated proteins evidenced following *B. terrestris* exposure to the bacteria *S. marcescens (S. m.).* The percentage was calculated by dividing the number of the proteins found in each biological process or molecular function identified with OmicsBox software by the number of the total dysregulated proteins following bacterial challenge. The pie-charts were made with Microsoft Excel 2021.

To describe the role played by differentially expressed proteins (DEPs) in response to bacterial infections, the up/down-regulated proteins were searched for their involvement in biological and molecular events in the bumble bee (Figure 7, Figure 8 and Figure 9 and Table S4). These proteins were found to be involved in several biological processes (mainly metabolism), some of them shared between the different types of bacterial infections and others specific to the inducer. 

A total of 716 pathways were identified as impacted, in which at least one protein was involved. Of these pathways, 86 were common to the three bacterial infection conditions, 76 were common to two bacteria (three between *M. l.* and *S. m.* and 73 between *P. c. c.* and *S. m.*) (Table S5), and 306 were specific to a given bacterial strain (five for *M. l*, 150 for *P. c. c.*, and 151 for *S. m.*) (Table S6). The top 10 pathways identified in each experimental infection condition are presented in Table 1.

Among these pathways, the glycolysis/gluconeogenesis pathway (ID: ko00010) was highly impacted by the three infectious conditions. Details of the involved up-regulated proteins in this pathway are mentioned according to each pairwise comparison to the control (Figure S1). The corresponding IDs of the proteins are shown in Table S5. It has been reported that in order to survive and multiply, bacteria must exert specific control over genes required for adaptation and growth within specific environments. For example, studies on bovine small intestine contents evidenced the importance of gluconeogenic substrates as carbon and nitrogen sources for *Escherichia coli* [52], and gluconeogenesis was also reported as an essential metabolic pathway for the pathogenic Gram-negative bacterial strain Francisella in mice [53].

As mentioned above, *Pectobacterium carotovorum* subsp. *carotovorum* strongly stimulates the immune response. The Toll and Imd signaling pathways (ID: ko04624) were identified as being impacted following *P. c. c.* infection by the up-regulation of defensin 1 (accession D2XR05, FC = 5.05) (Figure S2). We observed up-regulation of defensin 1 and abaecin, results that are consistent with previously reported data. For example, Lourenço et al. (2018) studied the relationship between AMPs, specifically defensin 1, and the dorsal genes in *A. mellifera* [54]. They reported that following the injection of dorsal-targeting RNAi, a significant down-regulation of the *defensin* gene was observed. The NF-κB factor of the Imd pathway was responsible for the up-regulation of *abaecin* and *hymenoptaecin*, but did not up-regulate *defensin* [55]. They concluded that the Toll pathway regulates *defensin* expression in *A. mellifera*, contrary to *Drosophila*, where *defensin* was primarily observed to be regulated by the Imd pathway [56]. Moreover, Erler et al. investigated the dynamics of immune system gene expression upon bacterial challenge and wounding in *B. terrestris* using qPCR. They reported an up-regulation of genes coding for abaecin, defensin-1, and hymenoptaecin, suggesting a greater impact at the gene level [18]. We also observed that *S. marcescens* had a greater impact on bumble bee survival than the other bacterial strains investigated. For this reason, we were interested in the specific pathways linked to this bacterium (Table S6). These include the detoxification and stress response pathways linked to the immune system. Interestingly, three proteins were exclusively up-regulated following *S. marcescens* infection, namely thioredoxin (A0A6P3TZ43), peroxiredoxin 1 (A0A151I178), and superoxide dismutase (Cu-Zn) (Q103C4), which were found to be involved in the two pathways mentioned above. Thioredoxin, as part of the thioredoxin system, was shown to play a crucial role in redox-regulatory processes and in protecting the organism against oxidative stress in a few species of insects, such as *Drosophila*, *Bombyx mori*, and *A. cerana cerana* [57]. Moreover, Mucci et al. reported that cold stress induced a specific oxidant response in honey bees [58]. However, Zaluski and colleagues reported the down-regulation of peroxiredoxin 1 and superoxide dismutase (Cu-Zn) (SOD) in the honey bee head [59]. SOD is part of one of the first lines of defense against reactive oxygen species (ROS) generated in the mitochondria or cytoplasm. SOD converts superoxide radicals to oxygen and hydrogen peroxide, which are then broken down by catalases and peroxiredoxins such as peroxiredoxin 1 [60]. Increases in SOD isoforms [61] and glutatione (GSTs) enzymes are associated with increased insect resistance to pesticides [62,63]. Another protein of interest is the heat shock 70 kDa protein cognate 4 (A0A6P3DAQ2) that was found to be up-regulated following *P. c. c.* and *S. m* challenge, and the heat shock protein 83 (A0A6P8M357), up-regulated following after *S. m*. infection. Members of the heat shock proteins (Hsp 18, 23 pseudogene, 25, 70, and 90) were found to be up-regulated for cold survival during insect diapause [64]. In 2020, Wrońska et al. reported that Hsp 90, 70, 60, and 27 in *Galleria mellonella* hemolymph are affected by infection with *Conidiobolus coronatus* [65].

## 3. Materials and Methods

### 3.1. Chemicals and Reagents

Acetonitrile (ACN), formic acid (FA), methanol, and trifluoroacetic acid (TFA) were of LC-MS grade and purchased from Carlo-Erba Reagents (Val de Reuil, France). *Rapi*Gest™ SF surfactant was from Waters (Milford, MA, USA). Modified sequencing-grade trypsin (Promega Corporation, Madison, WI, USA) was used for protein digestion. α-Cyano-4-hydroxycinnamic acid (4-HCCA), 4-vinylpyridine (4-VP), dithiothreitol (DTT), and all other chemicals were purchased from Sigma-Aldrich (St. Louis, MO, USA). All solvents, culture media, and buffers were prepared using 18.2 MΩ water purified with a Milli-Q system from Millipore (Bedford, MA, USA), herein referred to as ultrapure water.

### 3.2. Bacterial Strains used for the Experimental Infections

**Figure 10:**
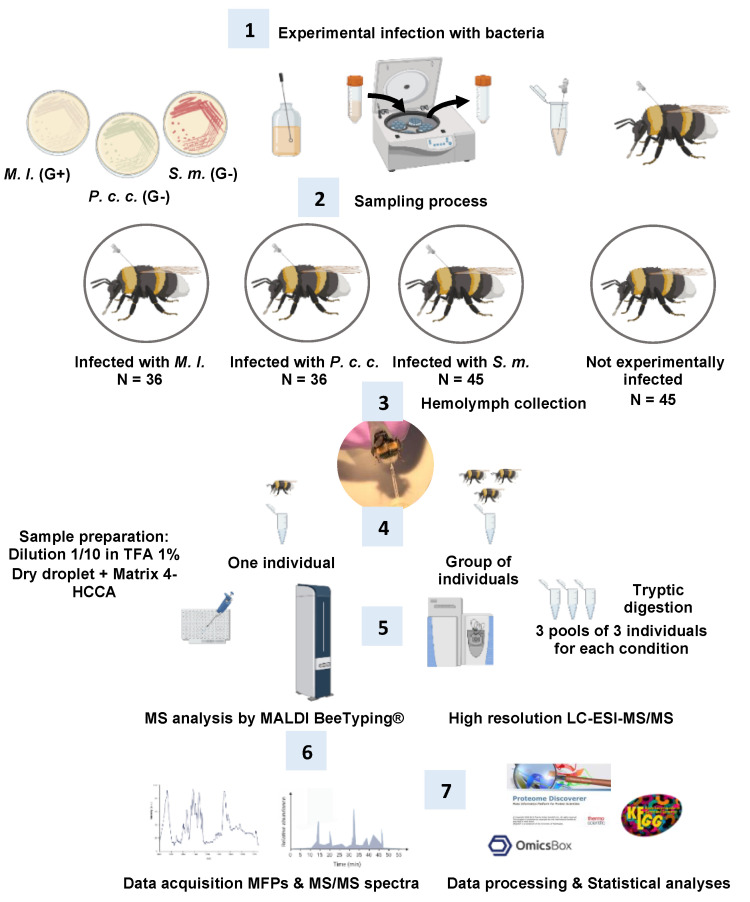
Omics workflow (Seven steps from experimental infection to data processing) applied to *Bombus terrestris*.

To generate biological models of bacterial infection, two entomopathogenic Gram-negative strains were used: (1) *Pectobacterium carotovorum* subsp. *carotovorum* 15 (*P. c. c.*, formerly called *Erwinia carotovora* subsp. *carotovora* 15 CFBP2141, a generous gift from Bruno Lemaitre, EPFL Switzerland) and (2) *Serratia marcescens* (*S. m.*, *Sm*BIOP160412, from our own collection). *Sm*BIOP160412 was an isolate from the haemocoel of a naturally infected *Apis mellifera* collected in the field. Bumble bees were also challenged with the Gram-positive *Micrococcus luteus* (*M. l.*, ATCC 4698), an opportunistic pathogen widely distributed in nature. Bacteria were cultured in Luria Bertani (LB) medium overnight at 32 °C (Figure 10, step 1).

### 3.3. Bumble Bees, Infection Experiments, and Hemolymph Collection (Figure 10, Steps 2 and 3)

#### 3.3.1. Bumble Bee Colonies

*Bombus* spp. are considered by the European Food Safety Authority (EFSA), along with *Apis mellifera* and solitary bees, as key model organisms in the risk assessment of plant protection products on bees. Three *Bombus terrestris* colonies were obtained from HELIOGREEN SAS (Fillinges, France) and maintained at room temperature (24 °C) continuously in the dark.

#### 3.3.2. Bacterial Infections

Experimental infections were performed according to a procedure detailed by Arafah et al. [29], with minor adjustments. Briefly, a group of 45 non-experimentally infected individuals were used as control, and three groups of individuals were infected with bacteria: 36 individuals were infected with *M. l.*, 36 with *P. c. c.*, and 45 with *S. m.* All individuals had approximately the same body size. Infections were performed by pricking the bumble bees individually in the thorax at the anterior lateral level under the wing with a fine needle (Fine Science Tools, Germany). The needle (0.1 mm in diameter, size used in general to infect small insects such as *Drosophila melanogaster*) was dipped into a concentrated pellet of live bacteria. This type of needle was used to minimize the invasiveness of the pricking and to reduce the size of the scar for rapid healing. All bumble bees belonging to the same group (experimentally infected and controls) were placed for 24 h at room temperature in small cages and fed ad libitum with sugar syrup (Invertbee from SARL Isnard, France).

#### 3.3.3. Hemolymph Collection

Hemolymph was collected from the dorsal side of the abdomen using pulled glass capillaries (Sutter Instrument Corp, Novato, CA, USA) following the procedure reported by Arafah et al. [29]. After collection, the hemolymph was immediately transferred to a chilled LoBind Protein microtube (Eppendorf, Germany) and pre-coated with phenylthiourea (PTU) and phenylmethylsulphonyl fluoride (PMSF) to prevent melanization and proteolysis, respectively. The hemolymph samples were frozen and stored at −20 °C until use (Figure 10, step 3, hemolymph collection).

#### 3.3.4. Survival Rate Assessment

The effectiveness of the experimental infection was evaluated by recording mortality. Mortality assessments were conducted before and at several time points post-experimental infections: at 6, 12, 24, and 48 h for the three types of infections, up to 72 h for the infection with *P. c. c.*, and finally up to day 6 for the infection with *M. l.* and for control samples.

### 3.4. MALDI Molecular Mass Fingerprints or MALDI BeeTyping^®^ (Figure 10, Steps 4–6)

#### 3.4.1. Sample Preparation

Before MALDI-TOF analysis, the samples were thawed on ice and then centrifuged. A tenfold dilution was performed by adding 0.5 µL of bumble bee hemolymph to 4.5 µL of a 1% solution of TFA in a 0.5 mL LoBind Eppendorf^®^ tube. For MALDI-MS analysis, the procedure used follows that of Houdelet et al. [36], with a minor modification. Briefly, the 10 fold diluted samples were deposited in three replicates on a MALDI plate (MTP 384 target plate polished steel BC, Bruker Daltonics) and vacuum-dried for 10 min. Once dried, the samples were covered with 1 μL of a fresh matrix solution (15 mg/mL 4-HCCA in 70% ACN, 2.5% TFA). Finally, the sample spots were lightly vacuum-dried before analysis. Calibration was carried out using 0.5 µL of APISCAL and 0.5 µL of Pepmix (Peptide Calibration Standard II, 700–3200 Da, Bruker Daltonics). APISCAL is an in-house calibration solution composed of two antimicrobial peptides from *Apis mellifera*, namely apidaecin (average *m*/*z* of 2109) and abaecin (average *m*/*z* of 3879); melittin (average *m*/*z* of 2847), the major venom component; and ETD151 (average *m*/*z* of 4839), a recombinant peptide. After drying under vacuum, the calibrants were covered with 1 μL of matrix. The plate was dried again before MALDI-TOF analysis.

#### 3.4.2. Sample Analysis and Data Acquisition

Mass spectra were acquired in triplicate on a MALDI-TOF AutoFlex III Smartbeam instrument (Bruker Daltonics) using the FlexControl 4.0 software (Bruker Daltonics) in an automatic positive linear mode. The instrument was set up with the following parameters: 200 Hz laser at a 50% global attenuation offset, an accelerating voltage of 1.3 kV in source, 9.25 kV lens voltage, 1.906 kV linear detector voltage, 120 ns of pulsed ion extraction delay, and 600 Da detector gating. MALDI-MS spectra were recorded at the mass range of 600–18,000 in *m*/*z* by summing 1000 laser shots. Data were previewed using the FlexAnalysis 3.4 software.

#### 3.4.3. MALDI Data Processing

MALDI data were imported into ClinProTools™ 2.2 software (Bruker Daltonics) for reprocessing. All spectra underwent baseline correction performed with a TopHat baseline algorithm, along with smoothing according to the Savitzky–Golay algorithm (window size 2.0 *m*/*z* in 5 cycles). The total average of the spectra was calculated based on a signal-to-noise threshold of 3 for peak selection, a picking height of 80, and an application of baseline. Peak lists (maximum peak number of 100) of each spectrum were extracted for data processing and statistical analyses. Comparative analyses were carried out between the different experimental conditions depending on the intensity of the selected peaks. The software normalized the spectra before performing statistical principal component analyses (PCA). Using PCA, we analyzed and compared the three characterized molecules specific to immunity in bumble bees, apidaecin (accession C0HKX3, average *m*/*z* of 1978), abaecin (accession D2XR04, average *m*/*z* of 4397), and defensin 1 (considering cysteine pairing), and a chymotrypsin inhibitor (AMCI, accession numbers K7WRE1, average *m*/*z* of 5938 considering cysteine pairing and an N-terminal pyroglutamic acid).

### 3.5. Bottom-Up Proteomics: Off-Gel Digestion and LC-ESI-MS/MS (Figure 10, Steps 5–7)

#### 3.5.1. Sample Preparation

Before proteomic analysis, only hemolymph samples collected at 24 h post-infection were grouped into three pools of three individuals for the control condition and the *M. l.* and *P. c. c*. infections, and into three pools of four individuals for the *S. m.* infection. The samples were then dried by vacuum centrifugation before being analyzed by a bottom-up proteomic approach following the method of Houdelet et al. [36]. Briefly, hemolymph samples were suspended in 0.1% RapiGest surfactant in 50 mM ammonium bicarbonate. After reduction with DTT at 56 °C for 30 min in the dark and alkylation with 4-VP at room temperature for 30 min in the dark, samples were digested overnight at 37 °C with 0.5 µg of trypsin. Digested samples were acidified with 5 µL of 20% ACN/10% TFA to stop enzymatic digestion. After 45 min of incubation at 37 °C, samples were centrifuged for 10 min at 15,000× *g* and transferred into glass vial inserts for further separation of the digested sample by nanoflow high-performance liquid chromatography (nano-HPLC).

#### 3.5.2. Nano-LC-MS/MS Analysis

Nano-LC-MS/MS was carried out using an Ultimate 3000 nano-HPLC (Thermo Scientific, Bremen, Germany) to separate the tryptic peptides according to the protocol established by Masson et al. [66]. The separation was performed on a reverse-phase column (3 µm, 75 μm × 250 mm), Acclaim C_18_ PepMap 100 from Thermo Fischer Scientific, using a biphasic linear gradient (water/ACN, each supplemented with 0.1% formic acid) from 2% to 32% ACN in 100 min and from 32% to 65% ACN in 5 min. A Q-Exactive mass spectrometer, equipped with a nanospray ion source, was used in positive mode and data-dependent acquisition. The voltage applied to the nanotips was adjusted to produce 0.3 μA and the entrance to the capillary was maintained at 320 °C. The Q-Exactive Orbitrap acquired a full-range scan from 380 to 2000 *m*/*z* (70,000 resolution, automatic gain control (AGC) target 3 × 10^6^, and maximum ion trap time (IT) 200 ms), and then fragmented the top ten peptide ions in each cycle (17,500 resolution, AGC target 2 × 10^5^, maximum IT 100 ms, intensity threshold 4 × 10^4^, excluding charge-unassigned ions, normalized collision energy of 30). Parent ions were excluded from MS/MS for the next 15 s. The software Chromeleon Xpress and Xcalibur 2.2 were used to control the nano-HPLC and the mass spectrometer, respectively.

#### 3.5.3. Database Searching and Protein Identification

The Sequest HT search algorithm was run by Proteome Discoverer™ 2.5 (Thermo Fisher Scientific, Bremen, Germany, GmBH) to match the acquired MS/MS spectra to a database consisting of protein sequences of the hymenopteran order (including *B. terrestris*), a selection of bee pathogens, and the three bacteria used in the experiment downloaded from UniProtKB, on 1 April 2022. The sequences of common protein contaminants (e.g., human keratins) were also added to this database. The following parameters were used: trypsin digest with three maximum missed cleavages; six and 150 amino acids as the minimum and maximum peptide lengths, respectively; a tolerance of 10 ppm/0.02 Da for precursors and fragment ions, respectively; cysteine pyridyl-ethylation was set as a fixed modification; and C-terminal protein amidation, methionine, and tryptophan oxidation were set as variable modifications. The MS features were extracted from the chromatographic timeframe between 20 min and 132 min and the min./max. precursor masses were selected at 350/500 Da, respectively. The identification confidence was set at a false discovery rate of 1%. The target/decoy selection was based on concatenated mode and validation made on q-Value.

Regarding the peptide validation node, a retention time shift of 10 min, a mass tolerance of 10 ppm, and coarse mode were used for parameter tuning. Label-free quantification was performed following a chromatographic alignment by the Minora Feature Detector node (maximum trace length of 5, a signal to noise ratio of 3, and a maximum ΔRT of isotope pattern multiplets of 0.2 min). The precursor ions of unique and razor peptides were used to determine protein abundance, and normalization was performed using the total peptide amount. Statistical significance was determined using an ANOVA (individual proteins) test on summed abundances with Top 3 and a max. fold change value set to 100. *p*-values were calculated and found significant when below 0.05. Protein ratios of <0.5 or >2 in the different conditions compared to the not-experimentally infected controls were considered as significantly differentially expressed (DEPs). To complete the missing protein identity information, NCBI tBlastn was used. For functional annotation of the lists of DEPs generated from the LC-ESI-MS/MS analyses, the OmicsBox bioinformatic software (v2.1.14, accessed on 1 September 2022) was used. To get the most complete annotation labels, analyses were performed using the four cloud-powered algorithms (Blast, InterProScan, GO Mapping, and GO slim). Separate lists of DEPs of the pairwise comparisons (different bacteria vs. control) were loaded to investigate the biological processes and protein functions following bacterial challenges. A combined pathway analysis was performed on the annotated sequences (proteins) joining Reactome and KEGG to identify the enriched pathways. A top priority taxon (*D. melanogaster*) was applied.

### 3.6. Statistical Analyses

Statistical analyses were performed using RStudio 1.4 software. The normality and homogeneity of the variances of the data were tested and indicated by a *p*-value < 0.05 *, a *p*-value < 0.01 **, and a *p*-value < 0.001 ***.

## 4. Conclusions

In an effort to better understand the impact of environmental stressors on the health status of *B. terrestris*, we designed an experimental workflow to (i) limit invasiveness, (ii) promote a rapid healing, (iii) generate models of bacterial infections to promote the strongest immune response in the bumble bee, and (iv) develop mass spectrometry analyses of the hemolymph to follow the impact of these bacterial infections on the health status of the bumble bee. Hemolymph, the circulating fluid or “blood” of insects, was analyzed by MALDI BeeTyping^®^, which proved to be effective in assessing the health status of the honey bee. MALDI BeeTyping^®^ allowed us to generate molecular mass fingerprints (MFPs)/peptidome profiling on bumble bee hemolymph. This study allowed us to demonstrate that bumble bees react in a specific way to environmental stresses such as infections by opportunistic and entomopathogenic bacteria. The molecular modifications of the hemolymph following a bacterial attack allowed us to distinguish between “healthy” individuals (not experimentally infected) and experimentally infected ones. Several immunity peptides and the chymotrypsin inhibitor AMCI from *B. terrestris* were detected and quantified as effective markers to assess the health status of bumble bees from a simple “blood” test using the innovative MALDI BeeTyping^®^ technique. These results highlighted predictive models capable of establishing the health status of bumble bees following experimental biotic stress with bacteria. Thanks to our off-gel bottom-up proteomics analyses, we were able to study the impact of bacterial pathogens and characterize for the first time the proteomics profiles of the hemolymph of *B. terrestris* subjected to different experimental infections with different bacteria known to be promoters of a mild to strong or deleterious response to such infections. Using a label-free quantification proteomics step, we showed that bacterial infection significantly modulates immune proteins, as well as several other proteins such as those involved in insect metabolism, responses to stimuli, and energetic pathways.

## Figures and Tables

**Table 1:** Top 10 pathways impacted following bacterial infection by either *M. luteus* (*M. l.*), *P. carotovorum* subsp. *carotovorum* (*P. c. c.*), or *S. marcescens (S. m.*). #Seqs means the number of proteins. They were classified according to the corresponding number of DEPs in which they were identified as being involved.

Bacteria	Pathway	Pathway ID	#Seqs
*M. l.*	Glycolysis/gluconeogenesis	ko00010	7
*M. l.*	Glucose metabolism	R-DME-70326	5
*M. l.*	Metabolism of carbohydrates	R-DME-71387	5
*M. l.*	Gluconeogenesis	R-DME-70263	4
*M. l.*	Innate Immune System	R-DME-168249	4
*M. l.*	Glycolysis	R-DME-70171	4
*M. l.*	Methane metabolism	ko00680	4
*M. l.*	HIF-1 signaling pathway	ko04066	4
*M. l.*	Carbon fixation in photosynthetic organisms	ko00710	4
*M. l.*	Neutrophil degranulation	R-DME-6798695	3
*P. c. c.*	Glycolysis/Gluconeogenesis	ko00010	7
*P. c. c.*	Innate Immune System	R-DME-168249	6
*P. c. c.*	Metabolism of carbohydrates	R-DME-71387	5
*P. c. c.*	Glucose metabolism	R-DME-70326	5
*P. c. c.*	Gluconeogenesis	R-DME-70263	4
*P. c. c.*	Glycolysis	R-DME-70171	4
*P. c. c.*	Neutrophil degranulation	R-DME-6798695	4
*P. c. c.*	Methane metabolism	ko00680	4
*P. c. c.*	HIF-1 signaling pathway	ko04066	4
*P. c. c.*	Carbon fixation in photosynthetic organisms	ko00710	4
*S. m.*	Innate Immune System	R-DME-168249	10
*S. m.*	Metabolism of carbohydrates	R-DME-71387	8
*S. m.*	Glycolysis/Gluconeogenesis	ko00010	8
*S. m.*	Neutrophil degranulation	R-DME-6798695	7
*S. m.*	Carbon fixation in photosynthetic organisms	ko00710	7
*S. m.*	Platelet activation, signaling and aggregation	R-DME-76002	6
*S. m.*	Glucose metabolism	R-DME-70326	6
*S. m.*	Methane metabolism	ko00680	6
*S. m.*	Response to elevated platelet cytosolic Ca2+	R-DME-76005	5
*S. m.*	Gluconeogenesis	R-DME-70263	5

## Data Availability

The MALDI MS raw datasets have been deposited in Figshare repository (https://doi.org/10.6084/m9.figshare.21989141) and the bottom-up proteomics raw data files and results are available to the readers through the ProteomeXchange Consortium via the PRIDE database (https://doi.org/10.6019/PXD035224)).

## Supplementary Materials

The following supporting information can be downloaded at: https://www.mdpi.com/article/10.3390/ijms24054658/s1.
