# Supplementary material for: Unraveling the Bombus terrestris Hemolymph, an Indicator of the Immune Response to Microbial Infections, through Complementary Mass Spectrometry Approaches
Source: Int J Mol Sci. 2023 Feb 28;24(5):4658. doi: 10.3390/ijms24054658 (PMC10003634; doi:10.3390/ijms24054658)
Supplement: Supplementary file 1 [file ijms-24-04658-s001.zip › ijms-2199395 figure.pdf]

Supplementary Materials

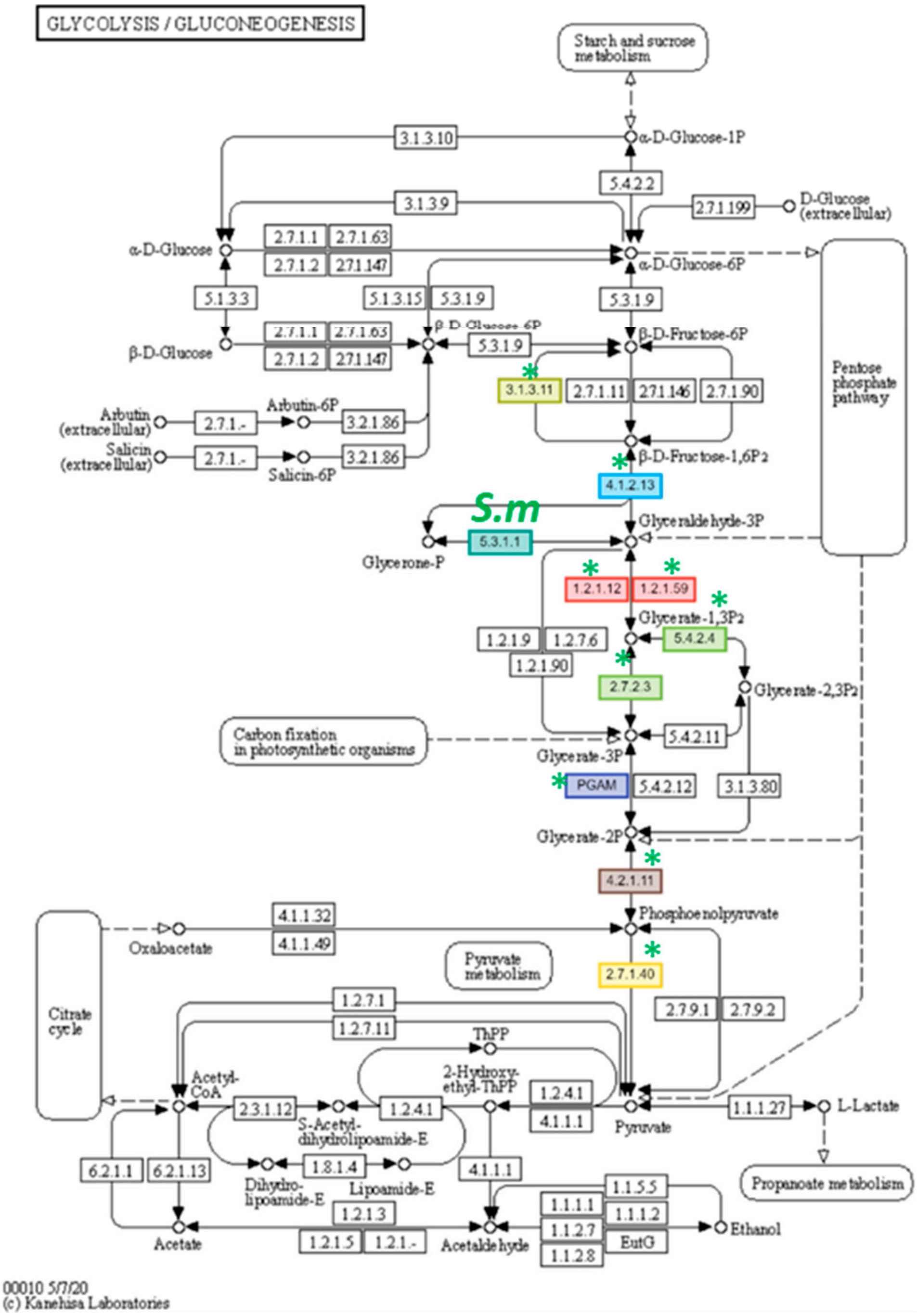

Figure S1: Glycolysis/Gluconeogenesis pathway with the involved up-regulated proteins following bacterial infection with *Micrococcus luteus* (*M. l.*), *Pectobacterium carotovorum* subsp. *carotovorum* (*P. c. c.*) and *Serratia marcescens* (*S. m.*). * means common to the three bacteria.

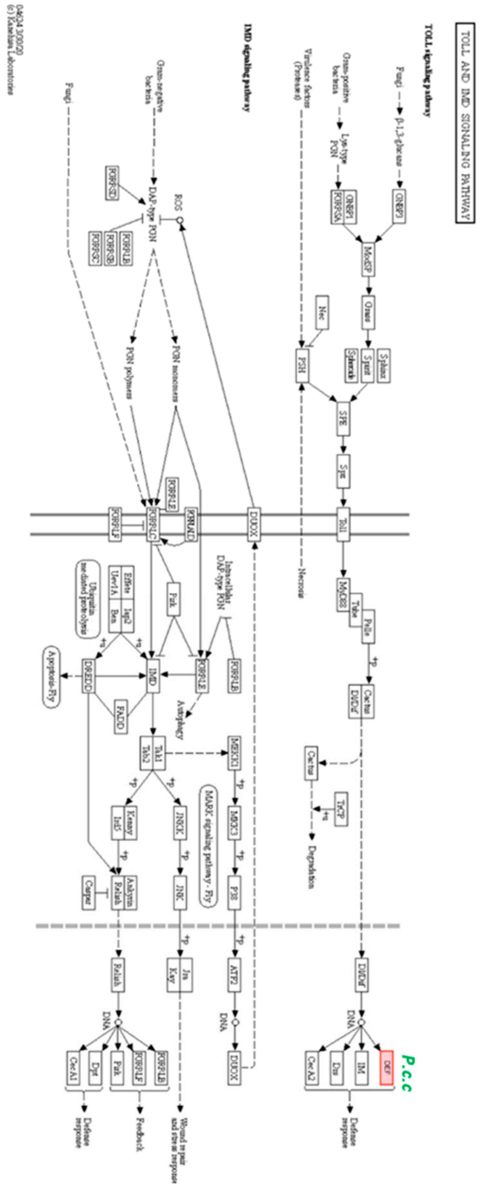

Figure S2: Toll and Imd signaling pathway impacted by *Pectobacterium carotovorum* subsp. *carotovorum* (*P. c. c.*) infection showing Defensin 1 (DEF).
